# Yaw Control Strategies Through Flow Structuring in Carangid C-Type Maneuvers

**DOI:** 10.3390/biomimetics11020156

**Published:** 2026-02-20

**Authors:** Yuansen Liu, Fei Li, Tianyu Gao, Shiyu Qian, Xiaolin Zheng, Yongliang Yu

**Affiliations:** 1 China Aerodynamics Research and Development Center, Mianyang 621000, China; lys614@mail.ustc.edu.cn (Y.L.);; 2 Lab for Biomechanics of Animal Locomotion, University of Chinese Academy of Sciences, Beijing 100049, China; 3 School of Ocean Engineering and Technology, Sun Yat-sen University, Zhuhai 519000, China; 4 Institute for Aero Engines, Tsinghua University, Beijing 100084, China

**Keywords:** yaw control strategies, flow structuring, C-type maneuvers, Carangidae fish

## Abstract

C-type maneuvers (abbreviated as C-turns), a crucial escape response from for carangiform fish, are investigated to elucidate their yaw control mechanism. High-speed photography coupled with image processing was used to quantify the time-varying midline curvature during C-turns of adult zebrafish (Danio rerio). Self-propelled simulations replicated the motion, resolving the evolving vorticity field and pressure gradients. Statistical analyses revealed a pronounced linear correlation between body deformation and total turning angle for yaw angles exceeding 60°. Notably, large-angle turns (>140°) exhibited both higher initial speed and sustained greater mean speed throughout the maneuver, indicating that achieving substantial yaw not only relies on enhanced body deformation, but also, critically, on inertial dominance persisting throughout the unsteady hydrodynamic interaction. The vortex dynamics and pressure distributions obtained form simulations corroborate the inferred control strategy rooted in this inertial dominance.

## 1. Introduction

Among aquatic organisms, fish are renowned for their precise control during swimming, exhibiting high maneuverability and low energy consumption [1]. As aquatic vertebrates with over 500 million years of evolutionary history, fish can be likened to precise biological machines. Under the precise regulation of neural signals, fish achieve undulatory motion by rhythmically contracting their muscles to drive the associated passive tissues like skin and vertebrae [2], converting stored biochemical energy into mechanical energy for propulsion. This process represents an optimal response to evolutionary pressures over millions of years [3]. The locomotion patterns formed to meet basic survival needs, such as predation, predator evasion, reproduction, and migration, provide important insights for the design of biomimetic propulsors.

In traditional metal or non-metal propeller-driven Autonomous Underwater Vehicles (AUVs), although inspired by fish, the biomimetic process partially loses the superior performance of fish [4,5] for the following reasons: 1. Difficulty in achieving omni-directional vector propulsion, making adaptation to complex deep-sea terrains challenging. 2. High navigation noise, easily disturbing the surrounding waters. 3. Insufficient response speed in low-speed environments. However, features such as low noise, high propulsion efficiency, and high maneuverability have broad application prospects in environmental assessment, seabed mapping, and scientific research assistance [6,7]. In-depth studies of fish high-maneuvering movements using experimental and numerical simulation techniques will undoubtedly inspire engineers to improve the efficiency and performance of biomimetic underwater propulsors [8,9,10].

With the advancement of experimental equipment and machine vision principles, capturing and reconstructing the three-dimensional motion of live fish has become increasingly accurate [11,12], meeting the requirements for biological motion simulation studies. In the field of Computational Fluid Dynamics (CFD), researchers have progressively developed a class of computational methods capable of simulating self-propelled motion, known as the Free Swimming Algorithm [13,14]. This approach couples fluid dynamics equations with rigid-body dynamics equations, enabling dynamic simulations of self-propelled systems such as biological propulsion and bionic robots.

Traditional hydrodynamic simulations of fish swimming usually use tethered models, where the airfoil is fixed in a uniform flow, and the fish body deformation is prescribed by given wave equations. However, in these simulations, fish body deformation is independent of fluid forces, and its position is unaffected by fluid forces and moments. Self-propelled algorithms, which solve motion equations to obtain the fish body’s position, have emerged to discuss kinematic characteristics (position, velocity, acceleration) under known deformation conditions. This approach ensures that the fish body is no longer constrained in a fixed flow, fully considering fluid–structure interaction (FSI) and maintaining momentum and angular momentum conservation throughout the fish’s motion [15]. For large deformation movements, particle methods (such as the immersed boundary method [16,17] and smoothed particle hydrodynamics [18]) are more robust than traditional CFD methods. The development of high-performance parallel processing and adaptive mesh refinement technologies has enabled in-depth studies of muscle work and skeletal structure performance under large deformation movements [19,20].

According to Breder’s classification system, fish swimming modes can be broadly divided into two categories: body and caudal fin (BCF) propulsion and median and paired fin (MPF) propulsion [21]. More than 88% of fish species rely on BCF propulsion for both locomotion and maneuvering [22,23], with Carangidae species being a prime example. Fish employing BCF propulsion typically exhibit superior maneuverability, enabling them to perform rapid, burst-like movements within a short time frame, achieving high levels of both translational and rotational motion. In contrast, MPF propulsion is characterized by its low energy consumption and high efficiency under the condition of relatively low velocity. Zebrafish, a species of Carangidae, also utilize BCF propulsion. Due to their ease of cultivation and size, zebrafish are frequently used as model organisms in studies of propulsion performance. Additionally, during the growth and development from juveniles to adults, zebrafish serve as a useful model for studying the transition from simpler sub-Carangidae swimming modes to the more advanced Carangidae mode.

Fast-start movements in fish can significantly alter their motion within a short time frame and are categorized into S-type faststart (S-start), C-type faststart (C-start), and C-type turning (C-turn), in which the latter two are collectively designated as C-type maneuvers. The S-start is typically associated with predatory behavior, with the northern pike’s hunting motion being a classic example. Both the C-start and C-turn are named after the “C” shape the fish’s body adopts during the preparatory phase, and they are further distinguished by their functional objectives. A C-start, which lasts less than 100 ms, is characterized by a rapid increase in translational velocity with limited rotational movement, primarily serving as a predator evasion tactic [24]. In contrast, a C-turn has a slightly longer duration (around 400 ms), allowing the fish to achieve a larger rotational angle. These type of maneuvers represents a compromise between swimming performance and energy efficiency, making them particularly suitable for investigating flow control strategies in fish locomotion [25].

The hydrodynamic mechanisms underlying fish C-type maneuvers trace back to the seminal work of Weihs [26], who first analyzed turning maneuvers using fluid dynamic principles, partitioning the fast-start into preparatory, propulsive, and coasting stages. The proposed added-mass model—where thrust generation arises from body-bending-induced fluid acceleration—remains the theoretical cornerstone for interpreting C-type maneuver dynamics. Experimentally, Domenici and Blake [27] established standardized performance metrics (turning angle, duration, peak angular velocity) across species, revealing that C-starts achieve angular velocities exceeding 3000 deg/s whereas C-turns operate at 1000 deg/s—a distinction critical for understanding their divergent hydrodynamic regimes.

Tytell and Lauder [28] employed time-resolved DPIV to resolve the three-dimensional vorticity evolution during Lepomis macrochirus escape responses, identifying three distinct jet structures (Jets 1–3) that correlate precisely with phase-specific body deformations. This work demonstrated that local vorticity generation along the body segments constructs coherent momentum jets through superposition. Numerically, Borazjani and Sotiropoulos [29] utilized the immersed boundary method to elucidate the effects of Reynolds number on carangiform swimming, revealing that body undulation increases drag in transitional regimes (Re ≈ 300), but reduces form drag in inertial regimes (Re ≈ 4000), providing crucial insights into velocity-dependent efficiency in C-type maneuvers. Gazzola et al. [30] further integrated evolutionary optimization with remeshed vortex methods, validating that hydrodynamic constraints dominate C-start kinematic selection and revealing an energy transduction pathway where preparatory-stage fluid entrainment (added-mass effect) is subsequently released as propulsive jet vortices.

Recent advances include Zhang et al.’s [31] vorticity-moment analysis of pressure gradient evolution during Carassius auratus turning, which identified strong negative-pressure zones on the concave body surface that transition to positive pressure as curvature waves propagate. Additionally, Liu et al. [32] demonstrated that large-angle C-turns (>140°) require the synergistic enhancement of initial velocity and deformation amplitude, establishing inertial dominance as a governing mechanism whereby elevated initial speeds augment inertial momentum and effective angle of attack for vorticity generation.

Despite these advances, critical gaps persist regarding the dynamic coupling among body deformation, initial swimming conditions, and flow structures. Specifically, (i) the partitioning of C-type deformation characteristics warrants deeper subdivision; (ii) a unified control framework encompassing both C-turns and C-starts remains elusive; and (iii) the spatiotemporal correlation between transient pressure fields and vorticity structures—particularly the antagonistic pressure pattern during the preparatory phase and its neuromuscular coupling—requires validation using self-propelled simulations.

This study investigates the control strategies and hydrodynamic mechanisms of yaw maneuvers in carangiform fish, using zebrafish C-type maneuvers as a model system. By integrating high-speed videography with self-propelled numerical simulations, we establish a three-phase analytical framework: (1) Experiment and Modeling: Morphological features of turning maneuvers were captured via high-speed imaging (Section 2.1), followed by geometric model reconstruction (Section 2.2). Autonomous propulsion simulations were implemented on the IBAMR platform (Section 2.3) and validated (Section 2.4). (2) Control Variable Identification: Time-varying curvature profiles of single maneuvers and statistical patterns from repeated trials revealed two critical control variables: C-shape deformation amplitude (Section 3.1) and initial maneuver velocity (Section 3.2). (3) Flow Field Analysis: Vorticity and pressure fields were analyzed to elucidate transient hydrodynamic structures (Section 3.3), force dynamics, and three-dimensional vorticity diffusion characteristics (Section 3.4) during maneuvers. The results demonstrate that large-angle yaw escapes in carangiform fish require synergistic coupling of body inertia and fluid viscous effects.

## 2. Materials and Methods

### 2.1. Capture of Fish Maneuvering Locomotion

**Figure 1:**
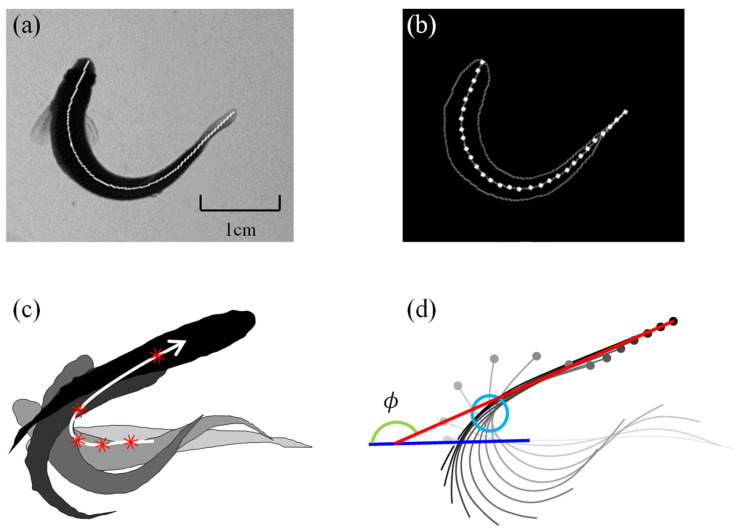
156.3° C-turn motion capture: (**a**) Experimental images. (**b**) Midline and contour in the binary image. (**c**) Contour, center of mass (red stars), and trajectory (white curve with arrows). (**d**) Midline information (grey curves, terminating in a circular dot denoting the anterior of fish), initial head direction (blue line), final head direction (red line), and total turning angle ϕ (green arc).

This study uses a motion capture platform based on high-speed photography technology to record the maneuvering motions of zebrafish under stimulation [32]. Zebrafish were group-housed in a tank at room temperature (20–25 °C) under a 12 h light/12 h dark photoperiod and fed daily at 9:00 a.m. prior to experiments. For the kinematic measurements, a healthy and active adult male zebrafish was selected and acclimated in the experimental tank for 2 h before being induced by acoustic stimuli to perform C-turn and C-start maneuvers. The experimental fish had a body length of *L* = 3.60 ± 0.14 cm and a body mass of *M* = 0.74 ± 0.09 g. By selecting adult male individuals with comparable sizes and physiological conditions, biological variability in body morphology and swimming performance was minimized in the kinematic analysis. Top-view high-speed cameras captured 8-bit grayscale images of these motions (as shown in Figure 1a).

Image processing techniques were employed to accurately identify the morphological characteristics of the fish. In the top-view images, the main features were the midline curve and the boundary contour of the fish body. First, appropriate thresholds were selected to binarize the 8-bit grayscale images based on the average grayscale difference between the captured image and the region containing the fish. Then, edge detection algorithms in mathematical morphology were used to identify the fish body contours, and thinning and skeletonization algorithms were combined to extract midline curve information from the binary images, as shown in Figure 1b. To analyze the control strategies and hydrodynamic mechanisms of large-angle C-turns, this study captured the morphological information of multiple C-maneuvers. Figure 1c shows the contour characteristics at different moments during a large-angle C-maneuver (progressing from light gray to black over time), the trajectory of the center of mass (white curve with arrows), and the center of mass positions at each moment (red stars). Following previous studies [33], the angle between the initial and final head directions during the maneuver was used as a criterion for characterizing the turn, identifying this motion as a 156.3° C-turn.

Figure 1d provides a detailed illustration of the midline posture at different moments, with solid circles indicating the head position and the midline transitioning from light gray to dark gray over time. The blue line represents the initial head direction, while the red line represents the final head direction, with the angle ϕ = 156.3° depicted by a green arc. Observations revealed that, for large-angle C-turns, the fish body deformation nearly passed through the same spatial location (marked with light blue circles). This phenomenon is due to the high energy conversion efficiency of carangiform fish’s large-angle C-turns, where the fish minimizes ineffective lateral motion to avoid energy loss and makes full use of the flow field structure formed in front. Further discussion on the hydrodynamic mechanisms behind this phenomenon will be presented in the following sections.

### 2.2. Simulation Geometric Model

**Figure 2:**
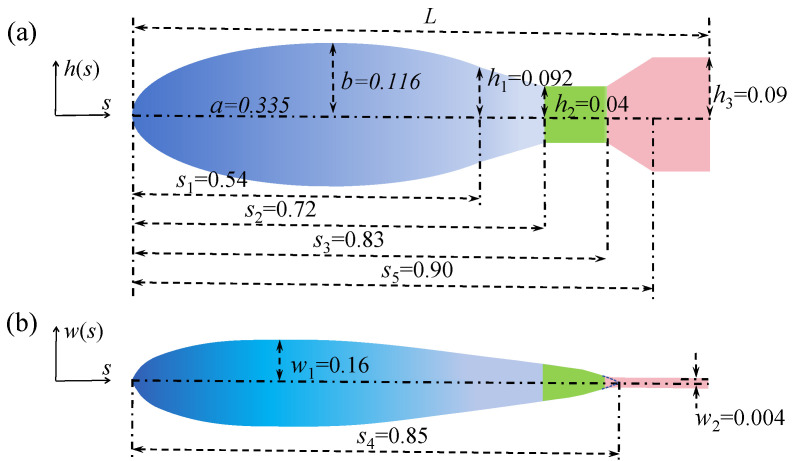
Geometric model of the fish body used in simulations: (**a**) Lateral view showing the height h(s). (**b**) Top view showing the width w(s).

Accurately capturing the geometric features of the fish body is crucial for simulating high-angle maneuvers. As shown in Figure 2, the Carangidae fish body consists of three main parts: the spindle-shaped torso (blue), the thin, plate-like caudal fin (pink), and the peduncle connecting them (green). The peduncle acts as a transition, with its cross-sectional shape gradually changing while its width decreases.

Although the zebrafish used in the experiments had vertical asymmetry in its geometric structure [32] (e.g., dorsal and ventral protrusions), this did not significantly alter the spindle shape of the torso. Additionally, fins (pectoral, dorsal, and anal) stabilize the fish during hovering and forward motion through small oscillations [34,35]. These fins contribute minimally to propulsion during Carangidae fish maneuvers [23] and are therefore omitted from the model. This study constructs a geometric model of the fish body based on the height *h*(*s*) in the lateral view and the width *w*(*s*) in the top view:

(1) $$h\left(s\right)=\left\{\begin{array}{cc} b\sqrt{1-{\left(\frac{s-a}{a}\right)}^{2}} & 0\leq s<s_{1},\\ h_{1}-\frac{h_{2}-h_{1}}{s_{2}-s_{1}}(s-s_{1}) & s_{1}\leq s<s_{2},\\ h_{2} & s_{2}\leq s<s_{3},\\ h_{2}-\frac{h_{3}-h_{2}}{s_{5}-s_{3}}(s-s_{3}) & s_{3}\leq s<s_{5},\\ h_{3} & s_{5}\leq s<L. \end{array}\right.$$

(2) $$w\left(s\right)=\left\{\begin{array}{cc} \frac{s_{4}w_{1}}{0.2}[0.2969\sqrt{\frac{s}{s_{4}L}}-0.1260\left(\frac{s}{s_{4}L}\right) & \\ -0.3516{\left(\frac{s}{s_{4}L}\right)}^{2}+0.2843{\left(\frac{s}{s_{4}L}\right)}^{3} & 0\leq s<s_{4},\\ -0.1015{\left(\frac{s}{s_{4}L}\right)}^{4}] & \\ w_{2} & s_{4}\leq s\leq L. \end{array}\right.$$

In the equations, *s* represents the arc length from any point on the midline to the fish’s head, normalized by the body length *L*. The parameters and their specific values are shown in Figure 2. The cross-sections of the torso and peduncle are symmetrical ellipses, with the maximum width *w*(*s*) modeled using the NACA0016 airfoil, and the caudal fin is modeled as a trapezoidal plate with a fixed width.

### 2.3. Numerical Methods

**Figure 3:**
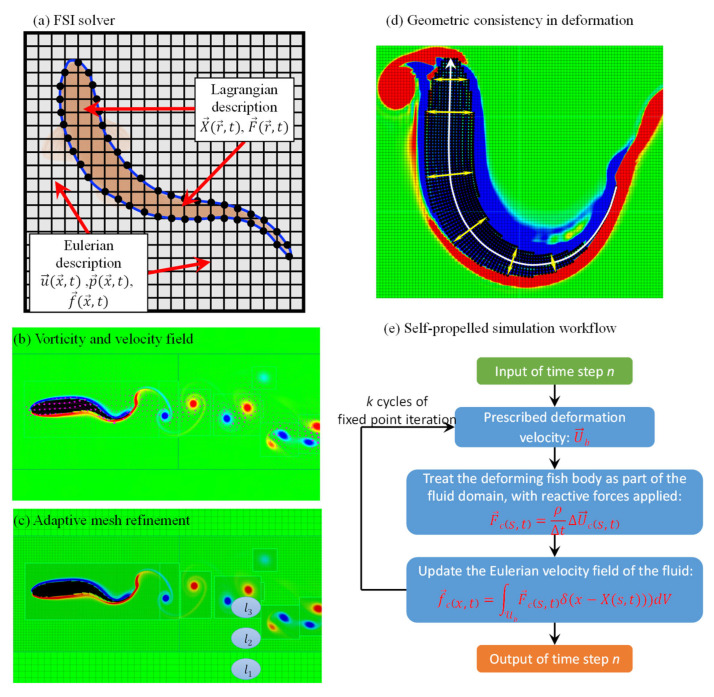
Using the immersed boundary method to study fish propulsion: (**a**) Implementation principle of the solver. (**b**) Vorticity and velocity fields obtained from cruise simulation. (**c**) Different levels of mesh refinement. (**d**) Geometric conservation during large deformations of the fish body. (**e**) Flowchart of the algorithm for the self-propelled solver.

The numerical simulations in this study were conducted using the constrained immersed boundary method [16]. As shown in Figure 3a, for solving such fluid–solid interaction (FSI) problems, a curvilinear grid partitioning was used, and the deformation of rigid and elastic bodies was handled using distributed Lagrange multiplier techniques. For the fluid domain, an adaptive Cartesian grid was employed, and the velocity, pressure, and force characteristics were solved using the Eulerian description. The governing equations for the computations are as follows:

(3) $$\frac{\partial \vec{u}\left(\vec{x},t\right)}{\partial t}+\vec{u}\left(\vec{x},t\right)\cdot \nabla \vec{u}\left(\vec{x},t\right)=-\frac{\nabla p\left(\vec{x},t\right)}{\rho }+\nu \nabla^{2}\vec{u}\left(\vec{x},t\right)+{\vec{f}}_{c}\left(\vec{x},t\right)$$

(4) $$\nabla \cdot \vec{u}\left(\vec{x},t\right)=0$$

(5) $${\vec{f}}_{c}\left(\vec{x},t\right)=\int_{u_{b}}{\vec{F}}_{c}\left(\vec{s},t\right)\delta \left(\vec{x}-\vec{X}\left(\vec{s},t\right)\right)dV$$

(6) $$\frac{\partial \vec{X}\left(\vec{s},t\right)}{\partial t}=\vec{U}\left(\vec{s},t\right)$$

(7) $$\vec{U}\left(\vec{s},t\right)=\int_{u_{b}}\vec{u}\left(\vec{x},t\right)\delta \left(\vec{x}-\vec{X}\left(\vec{s},t\right)\right)d\vec{x}$$

(8) $${\vec{F}}_{b}=\int_{\partial u_{b}}\sigma \cdot \vec{n}dA\left(\vec{x}\right)=M\frac{D{\vec{U}}_{b}}{Dt}-\int_{\partial u_{b}}{\vec{F}}_{c}dV$$

In these equations, $\vec{u}\left(\vec{x},t\right)$ denotes the velocity in the Eulerian description of the fluid, $\vec{p}\left(\vec{x},t\right)$ represents the pressure, ρ denotes the fluid density, and *µ* denotes the dynamic viscosity of the fluid. $\delta \left(\vec{x}\right)=\prod_{i=1}^{d}\delta \left(x_{i}\right)$ is the *d*-dimensional Dirac delta function used to interact the physical quantities of the fluid in the Eulerian description with those of the fish body in the Lagrangian description [36]. As shown in Figure 3b, in the cases used in this study, the rigid deformation of the fish body is assumed to be the same as the experimentally captured motion results, without considering the elastic properties of the structure. The body force ${\vec{f}}_{c}\left(\vec{x},t\right)$ acts within the region occupied by the fish body to impose constraints on parts of the object that exhibit prescribed motion or deformation kinematics. $\xi_{b}\left(t\right)$ denotes the region containing the fish body. In this framework, the pressure field $\vec{p}\left(\vec{x},t\right)$ is solved as part of the incompressible Navier–Stokes equations (Equations (3) and (4)) via a projection method: the velocity field is first advanced, then projected onto a divergence-free space by solving a pressure Poisson equation to enforce continuity. Spatial discretization uses a second-order accurate, staggered grid where pressure is stored at cell centers, enabling accurate computation of the pressure gradient $\nabla \vec{p}$ in the momentum equation. The adaptive mesh refinement (AMR) ensures adequate resolution in regions of strong pressure variation (e.g., near the body and in vortex cores). Surface pressure for force calculation is obtained by interpolating the Eulerian pressure field onto the Lagrangian boundary points using the regularized delta function $\delta \left(\vec{x}\right)$.

As shown in Figure 3a, a truncated fixed-point iteration method [37] was employed to accurately solve the motion and interaction of the fish body in free-swimming simulations. First, the deformed fish structure was treated as a fluid domain, where the deforming region of the fish is considered a special “fluid” rather than a rigid or elastic body. Constraints were applied within this fluid domain based on predefined equations, forcing the “fluid” to move according to the fish’s prescribed deformation [38,39]. This approach ensured that the dynamic deformations of the fish were transferred to the fluid, allowing for the fluid to follow the fish’s movement. To maintain the physical consistency of the entire “fish-water” system, corrections were made to the fluid velocity field at the fish’s location based on conservation of momentum and angular momentum principles. These corrections adjusted the flow characteristics around the fish to reflect its true dynamics. The truncated fixed-point iteration method then iteratively solved the system, adjusting the fish’s motion and forces based on the deformation and corrected fluid velocities until convergence was achieved. This approach effectively optimized the fish–fluid interaction, providing a high-fidelity simulation of autonomous swimming.

The open-source IBAMR code was used to spatially discretize the constraint governing equations [Equations (3)–(7)]. For multi-level Cartesian grids, Figure 3c demonstrates how the SAMRAI library was used for adaptive spatial refinement [40,41]. Near the grid resolution interfaces (i.e., coarse-fine interfaces), the finite difference approximations of the Eulerian spatial differential operators of the continuity equations maintained at least first-order accuracy locally. Far from the coarse–fine interfaces, the spatial discretization reverted to second-order accuracy staggered grid discretization. The cumulative hydrodynamic drag on the fish body was calculated using Equation (8), where the stress tensor is expressed as $\vec{\sigma }=-pΙ+\mu \left(\nabla_{h}\vec{u}+\nabla_{h}{\vec{u}}^{T}\right)$, where $\vec{n}$ is the outward unit normal vector on the surface of the immersed body and *M* denotes the mass of the immersed body. This method efficiently calculated the resultant force and torque on the fish body.

Following the reconstruction strategy proposed by Bhalla et al. [16], the experimentally measured midline curvature κ(s,t) was directly used to prescribe the instantaneous body configuration in the simulations. In this approach, the curvature information obtained from high-speed imaging serves as the primary kinematic input, from which the local body orientation and spatial configuration along the midline are reconstructed through successive integration along the body length. By adopting this procedure, the experimentally measured curvature data are transferred to the numerical framework without intermediate fitting or surrogate modeling. This ensures that the body geometry imposed in the self-propelled simulations is fully consistent with the measured kinematics at each instant, thereby establishing a direct and synchronized correspondence between experimental observations and simulation input.

### 2.4. Verification and Validation

**Figure 4:**
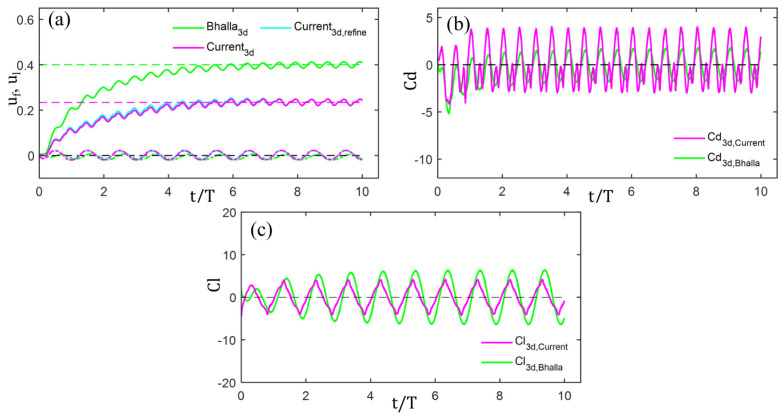
Comparative analysis of cruising motion data for different geometric shapes: Swimming speed is compared using the geometric models constructed by Bhalla and those developed in this study under (**a**) 3D conditions (the dashed line represents the mean cruising velocity under steady-state conditions). Additionally, comparisons are made of (**b**) the resistance experienced by the fish body and (**c**) the lateral forces.

To demonstrate the accuracy of the model and the domain selection, we performed model validation and grid independence tests. As shown in Figure 4, the proposed model shows decreased swimming performance compared to Bhalla’s eel example [16,17], mainly due to differences in zebrafish geometry and tail fin size, making the performance changes due to geometric differences physically consistent. Domain selection effects were assessed using adaptive grids with refinement ratios of 4*L* × 4*L* × 2*L* and 4*L* × 4*L* × 4*L*, where L is the fish length ($L_{fish}=3.60\;cm$), and vorticity thresholds of 0.25, 0.5, 1.0, and 2.0. Since computed forces are volume-dependent [Equations (3)–(7)], surface refinement minimally affects the computed forces in the absence of significant errors. Further grid refinement confirmed this finding. Therefore, to balance accuracy and computational cost, we use a periodic computational domain of size 4*L* × 4*L* × 2*L*. This domain employs four grid levels, with the coarsest level *l*_0_ having 16 × 16 × 8 grid cells. Levels *l*_1_ and *l*_2_ are refined by a factor of 4, and level *l*_3_ is refined by a factor of 2. To achieve as fine a boundary layer resolution as possible on the fluid–structure interface, the fish body is always kept in the finest grid level. When applying the AMR technique, the set vorticity thresholds are (1.0, 1.5, 2.0, 3.0), automatically triggering grid refinement when the vorticity in the fluid domain exceeds these thresholds. The convective CFL number is set to 0.1 to constrain the time step.

Finally, as shown in Figure 3d, to ensure geometric conservation during the simulation of large deformation maneuvers of the fish body, this study constructs the deformation characteristics of the fish body based on the midline curvature characteristics observed in experiments. Specifically, after obtaining the midline characteristics of the fish body through experimental and image processing work (white curve with arrows in the figure), each cross-section of the fish body is ensured to be perpendicular to the midline (yellow lines with double arrows in the figure). The midline curvature characteristics κ(*s*,*t*) in the body-fixed coordinate system are used to describe the maneuvering deformation of the fish body. This approach not only effectively captures the deformation characteristics, facilitating the analysis of flow control strategies during movement, but also perfectly fits the immersed boundary method, enabling precise simulation of the fish body’s maneuvering motion.

## 3. Results and Discussion

### 3.1. C-Shaped Deformation

This study investigates the control strategies of yaw maneuvers in Carangidae fish through integrated experimental observations and numerical simulations. Two critical control parameters were identified: C-shaped deformation amplitude and initial maneuver velocity. For distinct C-start maneuvers, the most significant variation observed experimentally lies in the deformation characteristics. To elucidate the influence of deformation on locomotion, we first present a representative 156.3° C-turn case, illustrating the correlation between body posture and deformation magnitude. Subsequently, differences in midline curvature for C-type maneuvers with varying deformation amplitudes are compared to derive the generalized patterns.

When characterizing C-type maneuvers, significant body deformation necessitates quantifying curvature dynamics in the head-fixed coordinate system for precise kinematic representation. Normalizing midline curvature κ(s,t) by body length *L* yields the deformation–posture relationship [42]:

(9) $$\begin{array}{c} \alpha \left(s,t\right)=\int_{0}^{s}\kappa \left(s,t\right)ds\\ \left(x\left(s,t\right),y\left(s,t\right)\right)=\int_{0}^{s}\left(\cos\left(\alpha \left(s,t\right)\right),\sin\left(\alpha \left(s,t\right)\right)\right)ds \end{array}$$

This normalization enables the cross-species comparison of Carangidae maneuvers. Experimentally, midline data preprocessing (interpolation and filtering) mitigates discretization artifacts in digital image analysis prior to curvature calculation [43]. In self-propelled simulations, curvature profiles are embedded into the motion file “IBEELKinematics3d.cpp” to accurately replicate body kinematics. As shown in Equation (9), the deflection angle α(s,t) at any midline point is obtained through integration, followed by coordinate transformation to derive positions (x(s,t),y(s,t)) in the head coordinate system.

**Figure 5:**
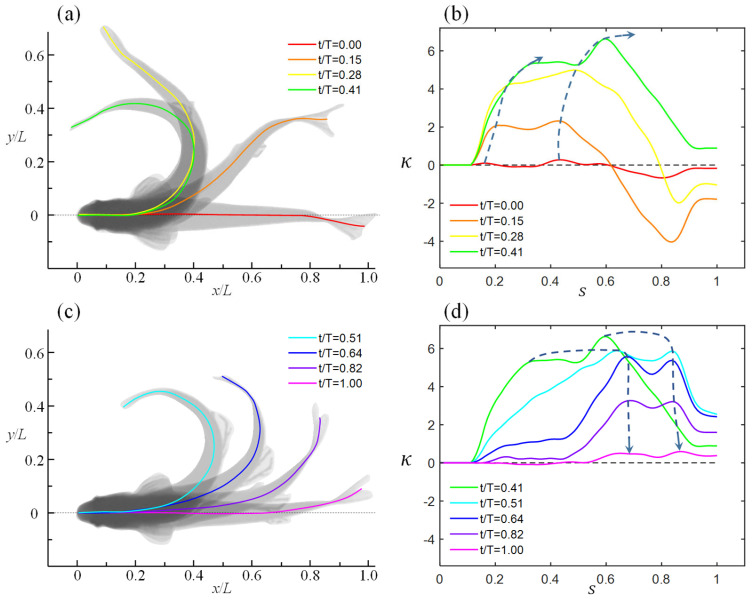
(**a**) Postures of the fish body at four characteristic moments during the bending phase. (**b**) Corresponding midline curvature at these moments (the black dashed lines represents κ=0). (**c**) Postures of the fish body at four characteristic moments during the recovery phase. (**d**) Corresponding midline curvature with the blue dashed line and arrows indicate the trends.

As depicted in Figure 5, C-type maneuvers are segmented into bending and recovery (swing back) phases based on the rotation direction. Subplots (a) and (b) illustrate the posture evolution and curvature dynamics at four characteristic timepoints during bending, while (c) and (d) display analogous data for the recovery phase. Blue dashed arrows annotate curvature trends at key locations, dynamically reflecting body oscillation properties. Timepoints are normalized by total maneuver duration (T=0.195 s) as $\frac{t}{T}$.

During the transition from gliding to bending phase, the midline curvature exhibits a “standing wave-like” elevation, with key characteristic values progressively increasing, indicating amplified body deformation. As time evolves, the standing wave curvature (annotated by blue dashed arrows) propagates posteriorly, transitioning into a traveling wave pattern. Notably, at $\frac{t}{T}=0.15$ and 0.28 (orange and yellow curvature profiles), the caudal peduncle-to-tail region moves opposite to the overall bending direction, demonstrating a counter-swing posture. This phenomenon arises from limited muscular capacity in the peduncle and passive deformation of the tail fin. Under strong fluid–body interactions, such counter-swing effectively enhances yaw propulsion.

In the return phase (Figure 5c,d), the body achieves forward propulsion via inertia after acquiring yaw angular velocity. Morphologically, the fish gradually straightens from its bent posture. Midline curvature initially displays traveling wave propagation, with key features shifting posteriorly during the return motion (blue dashed arrows in Figure 5d). Beyond $\frac{t}{T}=0.51$ (light-blue curve), corresponding to body straightening.

**Figure 6:**
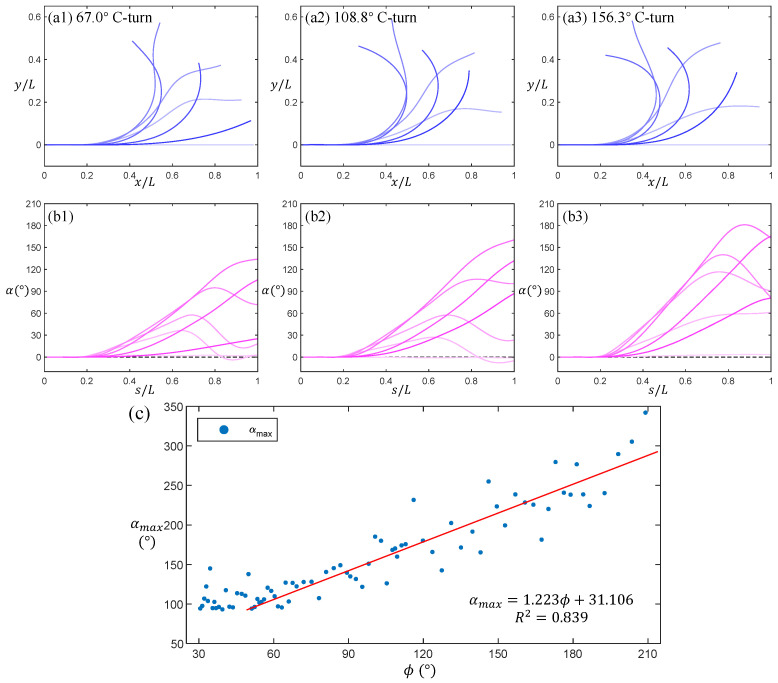
Deformation characteristics of C-turns with turning angles of (**a1**,**b1**) 67.0°, (**a2**,**b2**) 108.8°, and (**a3**,**b3**) 156.3° (blue curves) and the characteristics of the bending angle α(s,t) at different positions along the midline (pink curves). (**c**) The correlation between the maximum bending angle $\alpha_{\max}$ and the movement deflection angle ϕ in 80 datasets of C-turn maneuvers.

In order to validate this universality, 80 experimental trials of C-type maneuvers (rotation angles from 30° to 220°) were analyzed. Figure 6a1–a3 illustrates the deformation characteristics in the head frame for representative maneuvers at 67.0°, 108.8°, and 156.3° rotations. For each trial, one gliding, three bending, and three recovery phases were sampled. Temporal evolution is indicated by blue curves transitioning from light to dark. Quantitative analysis reveals that deformation amplitude scales positively with total rotation angle. Figure 6b1–b3 displays the bending angles α(s,t) derived from curvature integration (Equation (9)). Pink curves (light-to-dark gradient) temporally correspond to subfigures (a1–a3). These data visually demonstrate the spatiotemporal propagation of deformation features, elucidating the evolutionary mechanics of C-type maneuvers.

The maximum bending angle on the midline in the head-fixed coordinate system, $\alpha_{max}$, serves as an effective indicator of body deformation amplitude. Figure 6c plots the statistical distribution of $\alpha_{max}$ against the body rotation angle ϕ for 80 C-turn trials, revealing distinct patterns across angle ranges. For small-angle C-turns (ϕ∈[30°,60°]), $\alpha_{max}$ remains relatively constant with no significant dependence on ϕ. In contrast, for larger rotations (ϕ∈[60°,220°]), $\alpha_{max}$ exhibits a linear correlation with ϕ (the regression equation and coefficient of determination *R*^2^ are provided in the figure). Notably, within this larger angle range, the maximum midline curvature $\kappa_{\max}$ shows no significant variation, suggesting that while the maximum curvature is constrained by a physiological upper limit, fish achieve larger-angle deformation by engaging different body segments in the bending process. This explains why greater deformation amplitude (higher $\alpha_{max}$) results in larger rotation angles for C-turns.

These patterns are consistently observed in almost all yaw maneuvers of Carangidae fish, indicating that the C-turn is an efficient, evolutionarily optimized solution for rapid directional changes. The linear correlation between $\alpha_{max}$ and ϕ for large = angle turns, combined with the invariant $\kappa_{\max}$, highlights a biomechanical trade-off: fish maximize rotation angle by distributing bending across multiple body segments rather than exceeding the maximum curvature limit of a single segment.

In summary, the sequence “standing wave generation—traveling wave propagation—standing wave attenuation” provides a qualitative description of the deformation law for C-type maneuvers. For a successful C-turn, the bending phase is the core control strategy, which is characterized by controlled body deformation. Furthermore, the recovery phase, where the body gradually straightens via inertia, acts as an indispensable flow control mechanism to stabilize propulsion after the initial turn. Future studies could use sensitivity analysis to quantify how key kinematic parameters—such as midline curvature and timing—affect turning performance and vortex formation. Such parametric investigations provide a robust framework to clarify control variable importance and guide adaptive bio-inspired propulsion design [44].

### 3.2. Inertial Velocity of Maneuvers

In the C-turn maneuvers control of fish, the deformation parameter κ(s,t) is a critical factor. However, given the comparable densities of fish bodies and water, both body inertia (initial velocity) and fluid inertia (Reynolds number) play pivotal roles during maneuvers:

(10) $$Re=\frac{\rho UL}{\mu }=\frac{\rho L^{2}}{\mu T},\;\;u_{0}=\frac{U_{0}T}{L}.$$

The Reynolds number (Re) characterizes the relative importance of inertial to viscous forces in flow. Its value depends not only on the characteristic body length *L* and maneuver duration *T*, but also on fluid properties (density *ρ*, dynamic viscosity *μ*). For adult zebrafish performing C-turns, Re typically ranges between 1000 and 30,000. Additionally, the influence of body inertia cannot be overlooked. Physically, (1) deformation feature, curvature κ(s,t), reflects instantaneous body deformation; (2) fluid effect, Re, indicates viscous influence on maneuver dynamics; and (3) inertial contribution, initial velocity $u_{0}$, quantifies the inertial drive enabling yaw motion.

**Figure 7:**
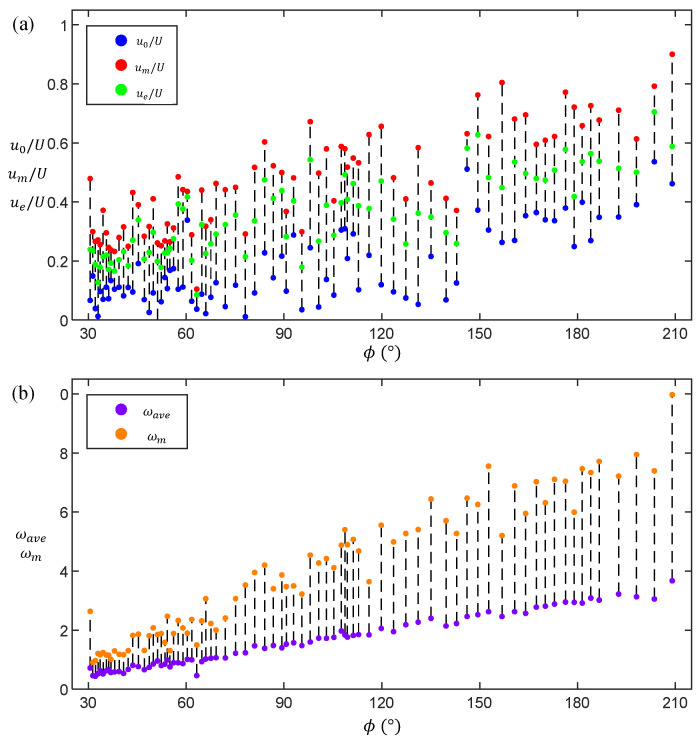
(**a**) Initial $u_{0}$, maximum $u_{m}$, and final velocities $u_{e}$ during C-turn maneuvers with varying turning angles from 80 trials, normalized by the characteristic velocity $U=\frac{L}{T}$; (**b**) average $\omega_{ave}\;$and maximum angular velocities $\omega_{m}$ of the fish body during the corresponding maneuvers.

Figure 7a statistically analyzes the translational features of 80 C-turns, displaying initial centroid velocity $u_{0}$ (blue circles), maximum velocity $u_{m}$ (red circles), and final velocity $u_{e}$ (green circles). All velocities are normalized by $U=\frac{L}{T}$ and connected by dashed lines. The results show that, for small yaw angles (ϕ∈[30°,140°]), translational velocity variations are minor, with generally low $u_{0}$ and slight post-maneuver acceleration. Here, 140° separates moderately from large-angle turns, consistent with fast-start studies identifying reorientation angles above 120° as distinct [27]. This reflects lower translational demands for small-angle yaws. For large-angle yaws (ϕ∈[140°,210°]), higher initial velocities $u_{0}$ are observed and substantial translational speeds persist throughout, indicating greater reliance on initial kinetic energy (inertia).

Figure 7b illustrates the angular velocity characteristics across yaw angles, including average angular velocity $\omega_{ave}$ (purple circles) and maximum angular velocity $\omega_{m}$ (yellow circles). Both correlate positively with total yaw angle ϕ. Given $\omega_{ave}=\frac{\phi }{T}$, the time scale T varies minimally across maneuvers, with key differences arising in achieved angular velocities (notably $\omega_{m}$).

In summary, within fluid environments of fixed physical properties, efficient large-angle yaw maneuvers in fish primarily rely on enhanced body deformation κ(s,t) and sufficient initial impulse $u_{0}$. The observed inertial dominance in large-angle C-turns is consistent with findings in other carangiform species of similar size, such as trout and sunfish, where initial body momentum plays a crucial role in enabling rapid directional changes during escape responses [27,45]. This cross-species similarity suggests that inertial-driven turning may be a conserved control strategy among fishes that rely on C-type maneuvers for predator evasion.

### 3.3. Flow Field Structure and Force Characteristics

Building on the experiments, the prior sections outline the key control strategies for C-turns. The subsequent sections employ self-propelled simulations to investigate flow field evolution, force mechanisms, and vorticity transport during maneuvers.

**Figure 8:**
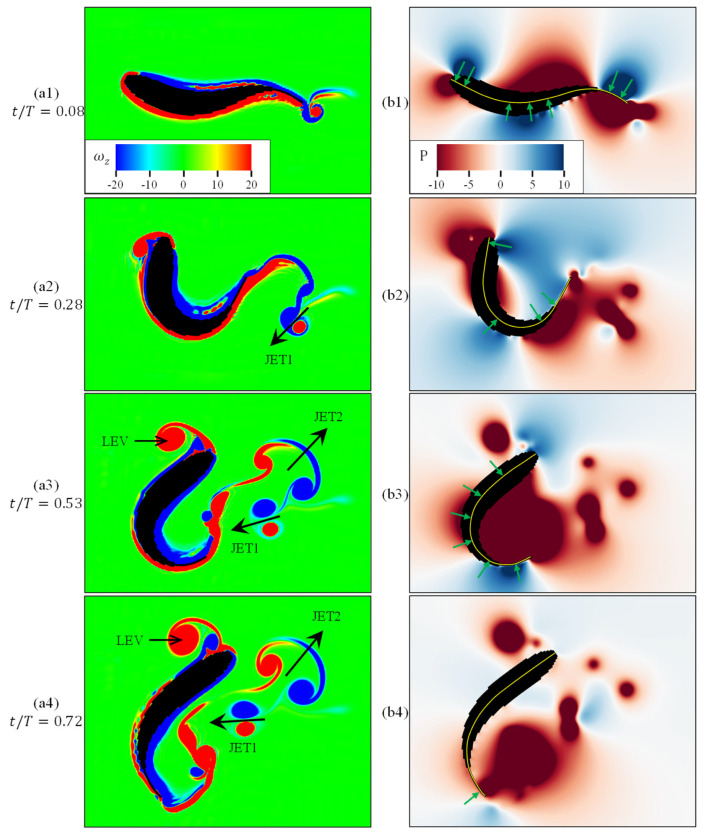
Vorticity fields $\omega_{z}$ and pressure fields P on the z=0 plane at various stages of fish swimming: start phase (**a1**,**b1**), bending phase (**a2**,**b2**), propulsion phase (**a3**,**b3**), and recovery phase (**a4**,**b4**). Black arrows denote the trailing jet, yellow lines represent the midline, and green arrows indicate the force.

Figure 8 presents the simulation results for a 156.3° C-turn, showing vorticity $\omega_{z}$ and pressure *P* fields at the z = 0 plane. The vorticity field reveals development of leading-edge vortices (LEVs) and tail-fin jets (JETs), while the pressure field visualizes the force distribution on the body surface. The added-mass force ${\vec{F}}_{add}$, arising from fluid momentum changes induced by body undulation [46], is expressed as

(11) $${\vec{F}}_{\mathrm{add}}=-\frac{D}{Dt}\left(m\vec{V}\right)$$

where $\vec{V}$ denotes lateral undulation velocity and m represents equivalent fluid added mass. Integrating this force over the body surface yields pressure-resultant forces whose forward projections contribute to propulsion efficiency.

The flow evolution and force mechanisms during the C-turn (Figure 8) are as follows, with the simulated vortex and jet structures corroborated by particle image velocimetry (PIV) experiments [47]:

**(1) Initiation phase** ($\frac{t}{T}=0.08$): Minimal body deformation and underdeveloped flow disturbances (a1–b1). A distinct pressure pattern emerges: the head and tail regions exhibit pressures aligned in one direction, while the mid-body shows opposing pressure. This antagonistic pressure distribution is a direct hydrodynamic signature of rapid white muscle contraction on one side of the body, actively curving the anterior section and initiating the C-shape posture. This active muscular input is crucial for setting the initial conditions that enhance subsequent rotational performance.

**(2) Deformation accumulation phase** ($\frac{t}{T}=0.28$): Body forms a C-shape (a2), inducing significant fluid momentum changes. LEV develops anteriorly, while primary jet (JET1) emerges posteriorly; both coincide with low-pressure zones (b2). The maintenance of this bent posture against increasing fluid loads requires sustained muscular contraction. The centroid velocity and direction remain stable during this phase, defining it as a preparatory stage where active body shaping stores energy in the fluid field.

**(3) Recovery phase** ($\frac{t}{T}=0.53$): A high-intensity jet (JET2) at the tail signifies the peak of fluid momentum exchange, driven by the rapid straightening of the body. The pressure field shows a synergistic pattern: a strong medial low-pressure region (associated with the jet) is flanked by lateral high-pressure zones (b3). This pressure gradient is the combined result of the tail’s active recoil (muscular action) and the inertial reaction of the accelerated fluid, which together drive a marked increase in translational velocity.

**(4) Extension phase** ($\frac{t}{T}=0.72$): LEV shedding continues, and jet structures dissipate viscously (a4). The fluid–structure coupling weakens, and the pressure field homogenizes (b4). The maneuver concludes with the fish gliding, with its motion now governed primarily by the inertia imparted during the earlier active phases.

Energy transfer pathways during C-turns involve muscle contraction overcoming viscous dissipation to input mechanical energy into the “fish-water” system, converting it to translational/rotational kinetic energy, vortex kinetic energy (LEV/JET), and pressure potential energy. Evolutionarily enhanced caudal fins and peduncles amplify energy transfer efficiency by increasing contact area.

### 3.4. Three-Dimensional Diffusion of Vorticity

During the execution of a C-type maneuver by Carangiform fish, the inertial interaction between the fish body and the fluid is undoubtedly significant, resulting in the dissipation of a portion of the energy. However, viscous forces also play a crucial role. Acting as a key linkage during the motion, they connect different kinematic phases and induce a series of secondary flow effects. Among these, the most prominent are the generation and evolution of jet structures and vortex rings. These phenomena not only influence the local dynamics of the flow field, but also significantly impact the propulsive efficiency and motion stability of the fish body.

Viscous effects exert a profound influence on the fish maneuvering process. Under the inertial-dominated motion mode, the final kinematic state of the fish body is closely related to the timing and amplitude of the tail recoil. To overcome drag, the fish relies on muscle contraction to generate torque for energy transfer. Concurrently, to move effectively by utilizing the fluid’s reactive force, structures such as fins optimize fluid interaction by increasing the contact area. However, under viscous effects, the drag experienced by the fish body also increases with contact area. The velocity attenuation caused by viscous forces inevitably limits the motion efficiency of the fish. Therefore, the fish needs to enhance its energy utilization efficiency by strategically leveraging flow field structures. For instance, flow structures induced by the motion of the anterior body segment (or anterior region) in a preceding phase can be effectively captured and utilized by the posterior segment (or posterior region) in a subsequent phase, thereby achieving energy recovery and redistribution.

**Figure 9:**
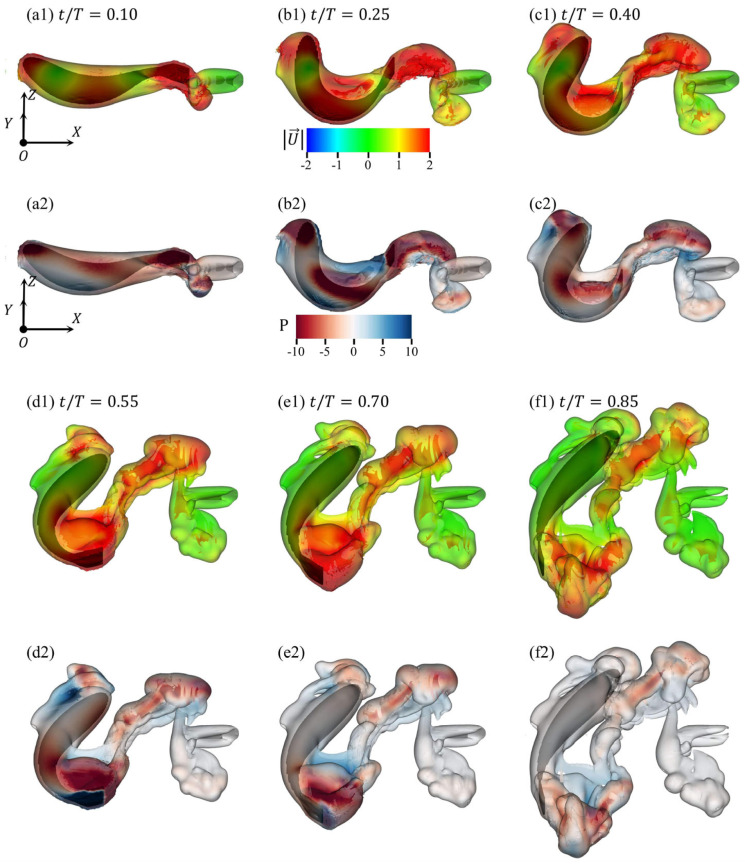
Flow visualization at $\frac{t}{T}$ = (**a1**,**a2**) 0.10, (**b1**,**b2**) 0.25, (**c1**,**c2**) 0.40, (**d1**,**d2**) 0.55, (**e1**,**e2**) 0.70, and (**f1**,**f2**) 0.85. The iso-surface of vorticity magnitude $\left|\vec{\omega }\right|=5$ is color-coded by velocity magnitude and pressure value.

To analyze the complex three-dimensional flow field characteristics generated by zebrafish maneuvering, Figure 9 presents the three-dimensional flow patterns and pressure values on the iso-surface of vorticity magnitude $\left|\vec{\omega }\right|=5$ at different characteristic time instants (expressed as the ratio of the instant to the total maneuver time T=0.224 s). The vorticity magnitude threshold ($\left|\vec{\omega }\right|=5)$ was selected to consistently capture the primary coherent vortex structures, such as the leading-edge vortex (LEV) and the tail-fin jet. The evolution and interaction of these vortices were quantified by tracking the spatial position and morphology of their iso-surfaces over time, providing a clear representation of vortex dynamics without explicit point-wise core tracking. The color coding for velocity magnitude and pressure value at points on the iso-surface is shown in the legends below Figure 9b1 and Figure 9b2, respectively.

During the body bending phase, the difference in the lateral forces acting on the body segments anterior and posterior to the center of mass causes a deflection in the direction of motion. Upon entering the recovery phase, the fish body continues to change its direction of motion due to inertia while simultaneously utilizing the reactive force from the fluid on the caudal peduncle and tail fin to maintain propulsion. During the maneuver, the fluid, subjected to the reactive impulse from the fish body, typically forms vortex ring structures that subsequently diffuse and evolve within the fluid. In large-angle yaw maneuvers, the fish body can even exploit disturbance structures formed by prior flow fields to minimize drag. As shown in Figure 9e1,f1, the fish body traverses the leading-edge separation vortex structure formed during the bending phase and closely follows the jet structure generated by tail oscillation during propulsion. This maneuvering strategy not only contributes to energy savings, but also enhances the stability of the entire yaw motion.

In an earlier study (Figure 8b1–b4), to clearly illustrate the fluid inertial forces acting on a fish during maneuvering, the vorticity and pressure characteristics were depicted within the z=0 plane. However, the complete three-dimensional simulated flow field clearly reveals that the so-called “positive and negative vortex pair” induced by the jet structure is actually the manifestation of a three-dimensional vortex ring structure on a specific cross-section. This interpretation aligns with experimental observations of fish swimming. For instance, Drucker and Lauder [48], using three-dimensional digital particle image velocimetry (DPIV), demonstrated that the pectoral fins of bluegill sunfish shed closed vortex rings during steady swimming, with the paired counter-rotating vortices observed in any two-dimensional plane representing slices through a coherent toroidal structure. Our simulations, while resolving the dominant vortex ring topology, inherently involve modeling simplifications such as prescribed body kinematics and rigid structural assumptions. These simplifications, though necessary for computational tractability, may not capture all of the subtleties of vortex ring connectivity or secondary three-dimensional instabilities present in the wake of a living, compliant fish. Nevertheless, the consistent identification of vortex rings as the primary wake structure across both high-fidelity simulations and experimental studies underscores the robustness of this hydrodynamic feature in fish locomotion. The diffusion process of this vortex ring proceeds as the jet develops throughout the flow domain, with the vorticity and pressure distributions gradually stabilizing over time. Therefore, throughout these C-type maneuvers, inertial effects primarily govern the kinematic performance of the fish body (such as velocity, turning angle, displacement), while viscous effects predominantly shape the vortical structures within the flow field and their evolutionary process.

In summary, the yaw maneuver of a fish body exemplifies a highly efficient utilization of fluid viscous effects by aquatic organisms. Viscosity exhibits a dual effect on fish propulsion: on the one hand, it continuously dissipates mechanical energy through vortex dissipation mechanisms; on the other hand, it provides the fish body with an additional source of thrust. By precisely regulating the flow field evolution and fully exploiting its structural characteristics (such as capturing leading-edge vortices and conforming to jet structures), fish significantly enhance swimming efficiency while effectively reducing energy loss. This flow field management strategy, culminating in the inertial-driven vortex ring of the C-turn, is specialized for rapid, large-angle reorientation. It differs from the hydrodynamic signatures of other common maneuvers, such as S-type starts or gradual turns, which may rely on more sequential vortex shedding and force continuity over a distributed spatial or longer temporal scale to serve different functions like predation or navigation [49]. This adept modulation exemplifies their high adaptability to their living environment. Building on these insights, machine learning approaches such as physics-informed neural networks (PINNs) may provide a promising means to further relate kinematic regulation to complex hydrodynamic outputs, including pressure fields and vortex dynamics [50].

From a biological perspective, the observed rapid yaw reorientation is consistent with the sensorimotor and neural control mechanisms reported in fish escape responses, where fast neural activation tightly couples body kinematics with flow-induced forces [27]. Moreover, yaw control strategies and the relative dominance of inertial versus viscous effects are known to vary with body size and swimming speed, placing the present findings within a broader ecological and scaling context [51]. The concentration of force production into inertial-driven vortex structures may also confer energetic advantages by enabling effective reorientation over short time scales, as suggested by prior studies on unsteady swimming efficiency [52].

## 4. Conclusions

This study systematically elucidates the control strategies and hydrodynamic mechanisms underlying C-type maneuvers in zebrafish (Danio rerio, a representative cyprinid species) using integrated high-speed photography experiments and self-propelled numerical simulations. The principal findings are summarized as follows:

(1) Kinematic and curvature characteristics: The midline curvature of the fish body exhibits a “two peak and one valley” mode. Characteristic curvature points delineate a three-phase dynamic process: initiation of standing waves, propagation of traveling waves, and attenuation of standing waves. This kinematic paradigm governs both C-start (translation-dominated) and C-turn (rotation-dominated) maneuvers.

(2) Core strategies of yaw maneuvers: Increasing body curvature amplitude enhances momentum transfer efficiency between the fish and surrounding fluid, while elevated initial velocity leverages body inertia to optimize directional changes. Their synergistic interaction significantly improves escape performance. Muscle contractions inject energy into the fish–fluid system via body deformation, with the caudal peduncle serving as the primary energy transduction module.

(3) Force dynamics and vortex mechanisms: Through the active modulation of transient vortex structures, zebrafish achieve dynamic equilibrium between viscous drag and propulsive gains via the synergistic effects of added-mass forces and vortex-induced forces. This hydrodynamic strategy maximizes energy utilization efficiency during maneuvering.

The closed-loop regulatory framework integrating “Deformation, Flow Field, Stress, and Kinematics” execution constitutes the cornerstone of zebrafish maneuver control. These insights offer three key principles for bio-inspired underwater vehicle design:

First, optimal maneuverability requires matching swimming speed ranges with context-appropriate body curvature amplitudes.

Second, vortex energy harvesting through caudal peduncle and fin structures enhances propulsion efficiency.

Third, phase synchronization between propulsor deformation and vortex field structures is critical during maneuver execution.

In practice, translating these principles to engineering applications requires consideration of several implementation challenges. Actuator bandwidth must be sufficient to replicate rapid and precisely timed body deformations, structural compliance should allow for energy storage and release without compromising stability, and control strategies must handle the coupled nonlinear dynamics between deformation, fluid forces, and vehicle motion. Acknowledging these factors highlights both the opportunities and limitations of applying zebrafish-inspired maneuvers to agile AUVs while emphasizing the value of biological insights in guiding design trade-offs.

## Data Availability

The data supporting the findings of this study are available from the authors upon reasonable request.

## Abbreviations

The following abbreviations are used in this manuscript:

AUV	Autonomous Underwater Vehicle
CFD	Computational Fluid Dynamics
FSI	fluid–structure interaction
BCF	body and caudal fin
MPF	median and paired fin
S-start	S-type faststart
C-start	C-type faststart
C-turn	C-type turning
LEV	leading-edge vortices
JET	tail-fin jets
